# The Case Against Exchange Transfusion Has Yet to Be Proved

**DOI:** 10.1093/cid/cit698

**Published:** 2014-01-15

**Authors:** Gavin C. K. W. Koh, Richard J. Maude

**Affiliations:** 1 Department of Infection and Tropical Medicine, Heartlands Hospital, Birmingham, United Kingdom; 2 Mahidol-Oxford Tropical Medicine Research Unit, Rajthewi, Bangkok, Thailand; 3 Centre for Tropical Medicine, Nuffield Department of Medicine, University of Oxford; 4 College of Medicine and Veterinary Medicine, University of Edinburgh, United Kingdom

Tan et al are to be congratulated for achieving the difficult task of synthesizing the many disparate pieces of data on the subject of exchange transfusion (ET) [1]. However, we believe their conclusion that “adjunct ET cannot be recommended” should have been qualified with an acknowledgment that they have shown a “lack of evidence for benefit” rather than “evidence for lack of benefit.” The reported nonsignificant detrimental effect of ET (odds ratio, 0.84) had a wide 95% confidence interval of .44–1.60 (crossing unity). As the authors mention in the discussion, there has been no adequately powered comparative trial for this intervention. The case for or against ET in severe malaria is not yet proven. We feel that a more appropriate conclusion would be that there is insufficient evidence either for or against ET and more research is needed, ideally in the form of a randomized controlled trial.
